# Myeloperoxidase as a therapeutic target for oxidative damage in Alzheimer’s disease

**DOI:** 10.1080/14756366.2025.2456282

**Published:** 2025-02-14

**Authors:** Astrid Mayleth Rivera Antonio, Itzia Irene Padilla Martínez, Mónica A. Torres-Ramos, Martha Cecilia Rosales-Hernández

**Affiliations:** ^a^ Laboratorio de Biofísica y Biocatálisis, Sección de Estudios de Posgrado e Investigación, Escuela Superior de Medicina, Instituto Politécnico Nacional, Plan de San Luis y Salvador Díaz Mirón s/n, MéxicoCiudad de México, México; ^b^ Laboratorio de Química Supramolecular y Nanociencias, Unidad Profesional Interdisciplinaria de Biotecnología, Instituto Politécnico Nacional, MéxicoCiudad de México, México; ^c^ Dirección de investigación del Instituto Nacional de Neurología y Neurocirugía Manuel Velasco Suárez. Av, Ciudad de México. C.P, México

**Keywords:** Myeloperoxidase, hypochlorous acid, Alzheimer’s disease, oxidative stress

## Abstract

Alzheimer’s disease (AD) is a major neurodegenerative disorder more common in older adults. One of the leading AD hypotheses involves the amyloid beta (A) production, it is associated to oxidative stress, neuroinflammation, and neurovascular damage. The interaction of A with the blood vessel wall contributes to the disruption of the blood-brain barrier (BBB), allowing neutrophil infiltration containing the myeloperoxidase enzyme (MPO), which produces hypochlorous acid (HOCl) a potent oxidant. Also, MPO could be released from the microglia cells and interact with the amyloid beta plaques. This review aims to study the role of MPO in the progression of AD, in particular its contribution to oxidative stress and neuroinflammation. Furthermore, to explore the MPO-potential as AD-biomarker to evaluate the therapeutic potential of its inhibitors to mitigate the neurotoxicity. Finally, revise MPO inhibitors that could act as dual inhibitors acting on MPO and acetylcholinesterase and or another target involved in AD.

## Introduction

Alzheimer’s disease (AD) is a neurodegenerative disorder characterised by amyloid beta (Aβ) deposits in the brain and the intracellular accumulation of neurofibrillary tangles of hyperphosphorylated tau protein (p-tau). AD most commonly occurs in people over 65 years of age and is one of the leading causes of dementia^1^. AD is related to oxidative stress (OS) generation, which involves the overproduction of free radicals such as superoxide anion (O_2·_ˉ), and oxidative molecules derived from oxygen, such as hydrogen peroxide (H_2_O_2_). There are enzymes involved in OS in the brain, such as NADPH oxidase, which catalysed the conversion of O_2·_ˉ to H_2_O_2_^2^. Myeloperoxidase (MPO) originate hypochlorous acid (HOCl)^2^. This is a powerful oxidant that acts as a chlorinating agent in different biomolecules; it is formed from the chloride anion (Clˉ) anion and H_2_O_2_^3^. H_2_O_2_ came also from the Aβ aggregates which reacting with metals such as iron, copper, and zinc yielding reactive oxygen species (ROS)^4–6^. MPO is involved in the development of AD, due to different markers generated by HOCl have been identified in these patients such as those from the tyrosine chlorination producing 3-chlorotyrosine, this is found elevated in the hippocampus^6^^,^^7^.

OS is associated with inflammation, especially when the inflammatory infiltrate is comprised mostly of neutrophils. These cells and macrophages contain MPO, it has been widely described its role during atherosclerosis, where MPO oxidised the low-density lipoprotein (LDL), the LDL oxidised are not bound to its receptor, then this are recognised by scavenger receptor on endothelial cells and by macrophages, forming the foam cell and impairing the endothelium. Then, MPO has been implicated not only in the cardiovascular disease where several information has been published^8^^,^^9^.

But also, in other diseases such as, cancers, renal diseases, lung diseases and neurodegenerative diseases^10^. In 2004 was published the mRNA-MPO expression in microglial and neuronal cells but principally surrounding the amyloid beta plaques in brain from AD patients. Also, the relation of neutrophils in brain capillary adhesion, blood brain barrier breaching has been described, proposing that this cell an important target in AD due to the identification of its upregulated genes^11^. Therefore, the neutrophil activation has been related to cognitive impaired^12^.

Several biomarkers related to the oxidised environment have been identified in AD patients some of them produced by MPO. Therefore, the use of antioxidants (AOs), which act by reducing reactive species, stimulating the enzymatic antioxidant system, or inhibiting prooxidant enzymes such as Glutathione peroxidase (GSH-Px)^13^, NADPH oxidase^14^ and MPO^15^, could provide an alternative for preventing or diminishing the effects produced by oxidant molecules like HOCl to improve AD treatment.

As a result, the evaluation of antioxidant molecules for Alzheimer’s disease (AD) that also inhibit MPO has been suggested. However, their effects are less potent compared to synthetic MPO inhibitors. Examples include tryptamine derivatives, rivastigmine and acylhydrazones which inhibit both MPO and AChE^14^^,^^16^^,^^17^; hydroxamic acids, which inhibit HDAC6 and possess antiaggregant and Aβ neuroprotective properties^18^.

Then, this review highlights the role of myeloperoxidase (MPO) in Alzheimer’s disease (AD), linking it to oxidative damage and neuroinflammation through the production of hypochlorous acid (HOCl). It explains how MPO, originating from infiltrating neutrophils and microglia, contributes to blood-brain barrier disruption and neurovascular damage. Furthermore, is discussed the role of MPO as AD biomarker and revised some MPO inhibitors which could be a therapeutic strategy to mitigate oxidative damage in this disease and mention those that could have dual activity acting as MPO and acetylcholinesterase inhibitors.

## The myeloperoxidase catalytic cycle

The peroxidase (EC 1.11.1.x) family is involved mainly in oxidation, innate immunity, hormone biosynthesis, and the pathogenesis of various diseases. The haem-peroxidase superfamily is classified in four subgroups according with their activities: (a) peroxidase-catalse, (b) peroxidase-cyclooxygenase, (c) peroxidase-chlorite dismutase and (d) peroxidase-peroxygenase. MPO, eosinophil peroxidase (EPO), lactoperoxidase (LPO), and thyroid peroxidase (TPO) are members of the peroxidase-cyclooxygenase subgroup^19^, and exhibit different functions depending on the organ, tissue, or cell type in which they are located^20^^,^^21^.

These peroxidases are characterised by their use of H_2_O_2_ as a substrate; although peroxidases play a key role in protective mechanisms, some peroxidases can also cause harmful reactions, such as the co-oxidation of endogenous substrates, drugs, and xenobiotics, which leads to the oxidation of lipoproteins, carcinogenesis and liver necrosis^21^^,^^22^.

Peroxidases are oxidoreductases containing a haem group as the active site and undergo a series of redox reactions when reacting with their substrates. The haem group of native peroxidases is usually ferriprotoporphyrin IX, which contains four pyrrole rings bound to a central Fe^+3^ cation. Depending on the human peroxidases this have different substrate specificities, redox properties, and interconversion kinetics of redox intermediates, despite sharing similar functional or structural homologies. In this review, we address the MPO enzyme^21–23^.

MPO is synthesised during myeloid cell differentiation; it is found in the azurophil granules of granulocytes, mainly neutrophils, and plays an important role in the immune response, eliminating pathogens such as viruses, bacteria, fungi and parasites^24–26^. MPO belong to the haem peroxidase superfamily and is the most important enzyme of the peroxidase‐cyclooxygenase subgroup. MPO is encoded by a gene located on the long arm of chromosome 17 (17q)^27^. This is a heterodimeric glycoprotein of 150 kDa which contain two light chins (15 kDa) and two heavy chains (60 kDa) with a structure 2/2^28^. The principal glycosylation occurs in a asparagine residues Asn322, Asn355, Asn391, Asn483, and Asn729^29^. In the protein data bank (PDB) exist 307 3D MPO structures, (when the myeloperoxidase word is introduced). The ID PDB 5QJ2 shown that MPO is a myeloperoxidase protein with a haem group bound covalently to three aminoacids from the protein chain, these interactions are responsible of the green colour of MPO, reason by which at the first MPO was called “verdoperoxidasa”^30^. Then, different interaction between these aminoacids with the haem group allow that the spectroscopic characteristics of the haem group change.

An important 3D structural characteristic of MPO is that it has over its surface a great amount of basic aminoacids residues such as lysine and arginine which at physiological pH form a cationic surface, able to interact with to the negatively charged amino acids of apoB‐100 of LDL producing its oxidation important process during the atherosclerosis. The surface-surface interaction between MPO-apoB-100 produce an increase of 90% in the MPO chlorination and peroxidation activities^27^^,^^31^.

After activation of neutrophils by a pathogen the nicotinamide adenine dinucleotide phosphate (NADPH) oxidase is activated and MPO is delivery in the phagosome and in the extracellular environment by degranulation or by association with neutrophil extracellular traps (NETs), inside of phagosome the superoxide anion (O_2_^.−^) is produced by the NADPH oxidase, the O_2_^.−^ is converted to H_2_O_2_ by the superoxide dismutase (SOD). Then, the H_2_O_2_ and halides (such as Cl^−^) are used as MPO substrate forming the MPO/H_2_O_2_/Cl^−^ system to produce the HOCl a powerful oxidant ^27^.

**Figure 1.**
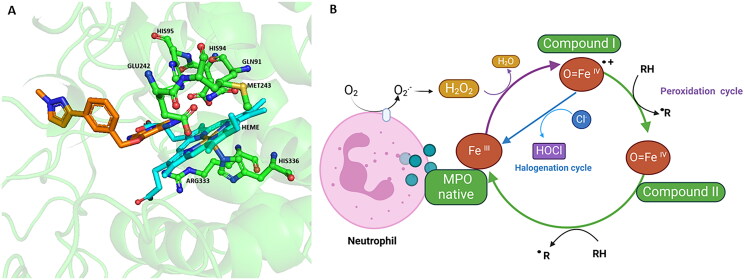
3D myeloperoxidase (MPO) structure and catalytic cycle. MPO enzyme has two catalytic cycles, peroxidation, and halogenation. (A) 3D MPO structure the haem group is showed and the interaction of these with the surrounding aminoacids. (B) Both cycles begins with the reaction of the native enzyme (Fe^III^) with hydrogen peroxide (H_2_O_2_), forming compound I [(Fe^IV^=O)^.−^](purple arrows). Compound I react with halides, mainly chlorides, forming HOCl and the MPO returned to its native state (Fe^III^). This is the halogenation cycle. Also, Compound I react with reducing agents (green arrow) to form Compound II (Fe^IV^=O) the most stable intermediate. Finally, the enzyme returns to its native state after reducing compound II (green arrow). This is the peroxidation cycle (green arrows). Figure created using Biorender software.

MPO is the only peroxidase capable of generating HOCl ^3^. In its native state or ferric form (Fe^+3^), MPO reacts with H_2_O_2_ to form compound I, which contains the ferryl haem specie [(Fe^IV^=O)^.−^]; this reaction (Figure 1; purple arrows) are part of the peroxidation (green arrow) and chlorination cycle (blue arrow), due to Compound I can reacts with halides (Brˉ, Clˉ, Iˉ and SCNˉ), mainly chloride, since this halogen is more abundant in the plasma, to form HOCl. After, MPO returns to its native state concluding the halogenation cycle (Figure 1; arrow blue)^18^. Therefore, in this cycle [MPO (Fe^+3^)] is converted to [MPO [(Fe^IV^=O)^.−^]] and finally returned to [MPO (Fe^+3^)]^18^.

But also the Compound I reacts with reducing substrates (RH) such as urate, tyrosine, tryptophan, sulfhydryl, nitric oxide, polyphenols, nitrite, xenobiotics and others, forming compound II [MPO (Fe^IV^=O)]which is the most stable form of MPO since it contains one less equivalent of oxidation than compound I (Figure 1: green arrow), which is subsequently reduced and returned to [MPO (Fe^+3^)] (Figure 1: green arrow). HOCl is capable of oxidising biomolecules such as proteins, DNA and lipids^3^, causing both oxidation and chlorination^23^. Thus, MPO plays a very important role in the onset and progression of chronic degenerative diseases such as Alzheimer’s disease, Parkinson’s disease (PD), cancer, and atherosclerosis, among others^25^^,^^26^^,^^32^^,^^33^.

## Oxidative stress (OS) and HOCl generated during AD

OS is characterised by an imbalance in the redox state, which can result from the production of an excess of reactive oxygen species (ROS) due to excessive oxidant enzyme activity, inactivation of antioxidant enzymes, or decreased production of antioxidants^34^^,^^35^. The brain consumes 20% of the total oxygen used by the human body, ten times more than other tissues do, which increases the probability of generating ROS, such as superoxide anion (O_2_ˉ) and H_2_O_2_. Furthermore, the brain has 50% of its dry weight of lipid content being the second tissue with the highest lipid content after adipose tissues^36^. Some of the brain lipids principally are cholesterol, phospholipids, such as phosphatidylcholine (PC) and phosphatidylethanolamine (PE), and sphingolipids then these lipids can be oxidised contributing to brain damage by the OS^37^.

Thus, the brain has its own antioxidant system that maintains a redox balance in brain cells, such as neurons and glial^38^^,^^39^. AD is associated with elevated ROS levels due to chronic inflammation, producing OS in neurons, which subsequently activates cell signalling pathways, such as stress-activated protein kinase (SAPK)^39^.

OS plays a principal role in AD progression, mainly through the oxidation of macromolecules such as lipids, and promotes the disruption of DNA–protein cross-links, oxidative cellular damage, the redox potential of Aβ^40^, mitochondrial dysfunction, and ROS generation, thereby positively regulating Aβ and p-tau production^41^.

Products obtained from lipoperoxidation include malondialdehyde (MDA), 4-hydroxy-2,3-nonenenal (4-HNE) and F2-isoprostanes; these are small and lipophilic intermediates capable of crossing the blood–brain barrier (BBB), reaching the bloodstream, and being used as markers of cerebral oxidation in AD, principally MDA and 4-HNE^42^. In addition, owing to their chemical reactivity, MDA and 4-HNE can stimulate p-tau phosphorylation, disrupt intracellular Ca^2+^ signalling pathways, and induce apoptosis. Furthermore, reactions such as hydroxylation, carbonylation and nitration can cause oxidative damage to both nuclear and mitochondrial DNA nucleotides, producing various biomarkers used to determine oxidative damage, such as the nucleotide 8-hydroxydeoxyguanine (8-OHdG)^14^^,^^41^.

In addition, the contribution of OS in different AD models has been evaluated; for example, intracerebroventricular administration of Aβ molecules of various lengths, such as Aβ_25–35_, in rats results in oxidative stress, a significant increase in the levels of the malondialdehyde (MDA) and *Mpo* genes, and a decrease in superoxide dismutase (SOD) levels. However, the administration of donepezil and *A. sinensis* optimal formula (AOF), a traditional medical herb in Asia, reversed the effects of Aβ_25-35_ administration^12^.

Furthermore, in an AD model induced by D-galactose (D-gal) and aluminium trichloride (AlCl_3_) in mice, an increase in OS was detected due to a decrease in enzymes such as SOD and GSH-Px and an increase in MDA in both the cortex and hippocampus. Perindopril administration diminished OS, which was accompanied by an increase in AO enzymes and a decrease in MDA^43^.

**Figure 2.**
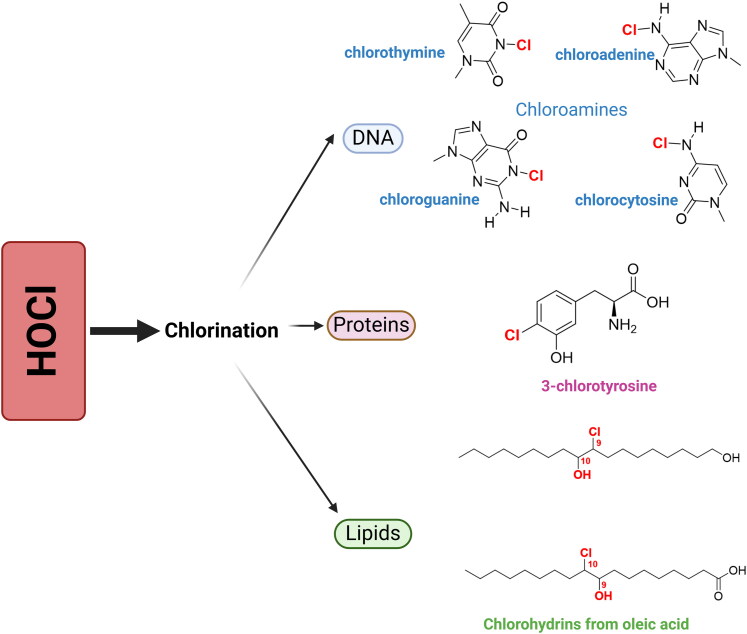
Hypochlorous acid (HOCl)-mediated chlorination of biomolecules. HOCl chlorinates the nitrogen bases in DNA to form chloramines. In proteins, HOCl chlorinates amino acid residues such as tyrosine to form 3-chlorotyrosine, and chlorination in lipids results in the formation of chlorohydrins. Figure created using Biorender software.

In addition, several biomarkers resulting from oxidation by HOCl are observed, such as chlorohydrins, which are produced by lipid oxidation when HOCl binds to the double bonds of unsaturated fatty acids and cholesterol (Figure 2)^44^. In DNA, HOCl causes structural changes and chemical modifications, forming semistable chloramines and causing DNA chain dissociation due to the disruption of hydrogen bonds (Figure 2)^45^.

Furthermore, the reactions of HOCl depend on the amino acid side chains, and both the primary amines and the sulphur-containing side chains (cysteine and methionine) are more prone to modification. Cysteine is chlorinated to form sulfenyl chloride, which then reacts with water and forms sulfenic acid^46^^,^^47^. Methionine results in the formation of stable methionine sulfoxides after being chlorinated and reacting with water^47^^,^^48^.

Other amino acids that undergo modifications by HOCl include lysine, histidine, and arginine, which react with the amines present in the side chains, leading to their mono-chlorination (N-chlorination), and potentially dichloramines. In tryptophan, HOCl reacts with indole, and tyrosine is halogenated within the aromatic ring; this modification allows HOCl to be widely used as a biomarker to detect HOCl acid-induced protein damage because of its specificity and high stability^49^.

3-Chlorotyrosine is formed from the chlorination of tyrosine residues (Figure 2), which is used as a biomarker of HOCl production, indicating that MPO is active in the brain^14^^,^^50^.

Rayner et al. (2014) revealed that HOCl can act as a cell signalling molecule by activating the extracellular signal-regulated mitogen-activated protein kinase (MAP) pathway (ERK1/2) within 2 min and activating the p38MAP kinase pathway within 15 min^51^.

HOCl is primarily removed by thiol-containing antioxidants, such as glutathione and taurine, although ascorbic acid and some phenols and hydroquinones can also reduce HOCl^52^^,^^53^. However, these antioxidants do not compete with the reactions that occur between HOCl and amino acids, peptides, and proteins because of their abundance and reactivity^54^.

The product obtained from the reaction between GSH and HOCl is glutathione disulphide (GSSG), and its turnover in the brain is very slow^52^. In addition, HOCl inactivates GSH peroxidase and GSSG reductase through the consumption of NADPH. Therefore, an increase in HOCl production can lead to the downstream generation of hydroxyl radical (·OH), nitrogen dioxide (NO_2_) and nytril chloride (NO_2_Cl) and cause cell damage via the oxidation, chlorination or nitration of biomolecules^54^.

As previously mentioned, HOCl shows high affinity for proteins. Thus, this reaction can modulate protein activity, such as in the activation of matrix metalloproteinases (MMPs), by oxidising a cysteine residue within the propeptide region. This modification subjects pro-MMPs to autocatalytic cleavage and allows activation of the zymogen^54^^,^^55^. However, matrix metalloproteinase-9 (MMP-9) inactivation also occurs at high HOCl concentrations^55^. Furthermore, HOCl can inhibit different enzymes some of them from the glycolytic pathway affecting the energetic metabolism such as glyceraldehyde-3-phosphate dehydrogenase, lactate dehydrogenase, hexokinase and other enzymes like creatine kinase, this effect also is due to the ability of HOCl to diffuses through the plasma membrane ^56^^,^^57^.

HOCl induces cell death by causing stress, and HOCl-mediated apoptosis involves caspase activation and mitochondrial permeability transition with concomitant release of cytochrome c. Furthermore, HOCl depletes the intracellular levels of ATP and GSH, and GSH depletion could play an important role in the degradation of the antiapoptotic protein Bcl-2. It also inhibits Ca^2+^-ATPase and activates Ca^2+^ release channels, increasing intracellular calcium levels^54^.

Wan et al. reported that HOCl induces apoptosis in cortical neurons in the absence of caspase activation and that the cell death process involves calpain activation and lysosomal rupture. In addition, increased cytosolic Ca^2+^ concentrations are associated with HOCl neurotoxicity^6^.

## MPO localisation in healthy individuals and AD patients

**Figure 3.**
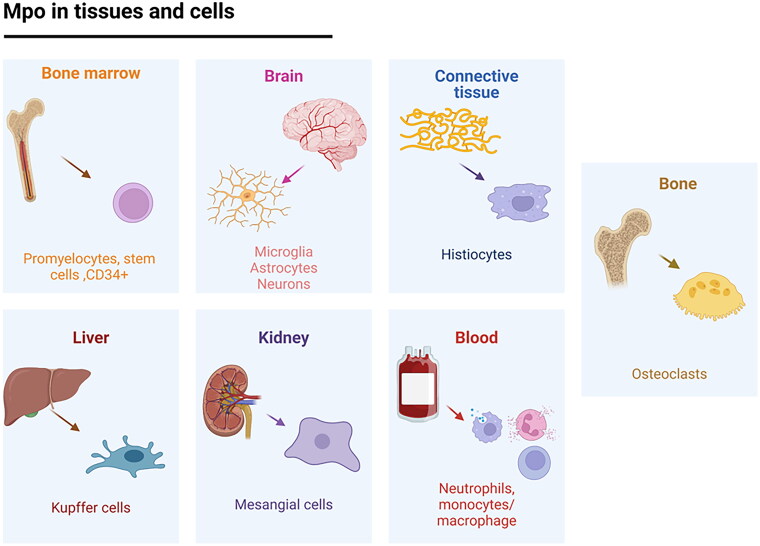
Myeloperoxidase expression in different cells and tissues.

MPO is present not only in neutrophils but also in monocytes; however, its concentration is up to 60% lower than that noted in neutrophils^58^. In tissues, monocytes differentiate into macrophages, decreasing their microbicidal activity. However, significant MPO levels are detected in various subpopulations of macrophages, such as Kupffer cells in the human liver^59^, alveolar macrophages^60^, macrophages in human atherosclerotic lesions^9^^,^^61^ and microglia in the central nervous system (CNS) (Figure 3).

Microglia are resident immune cells and constitute the first line of defense against pathogens in the CNS^62^^,^^63^. Microglia are activated by the presence of toxic substances, ageing, or foreign bodies produced in AD disease, such as senile plaques, and damaged neurons that produce inflammation in the brain. When the microglia cells are activated, they produce inflammatory mediators, such as cytokines, increasing ROS and RNS levels^32^^,^^39^. In addition, the Aβ peptide directly activates NADPH oxidase in microglia, producing O_2_^.−^and increasing H_2_O_2_ levels^39^.

The ability of macrophages to express MPO and generate HOCl *in vitro* is regulated by granulocyte–macrophage colony-stimulating factor (GM-CSF)^64^. MPO levels have also been studied in cells throughout the inflammatory process, increasing its levels in the early stage of inflammation in neutrophils (1–3 days), whereas in macrophages/microglia, MPO was more abundant thereafter (3–7 days)^65^. MPO expression has been identified in different tissues and organs during different disease states (Table 1).

**Table 1.** Myeloperoxidase (MPO) can be found in different tissue organ and cells.

Tissue or organ	Cells	Diseases
Bone marrow	Promyelocytes, stem cells, CD34+^66^	Cancer
Liver	Kupffer cells	Liver diseases^59^ Non-alcoholic steatohepatitis^67^
Connective tissue	Histiocytes	Kikuchi’s lymphadenitis^68^ Subacute necrotising lymphadenitis^69^
Kidney	Neutrophils, mesangial cells	Nephrotic syndrome^70^
Bone	Osteoclasts	Bone homeostasis^71^
Blood	Neutrophils, monocytes/macrophage	Atherosclerosis^9^
Brain	Neutrophils, activated microglia, monocytes/macrophage, neurons, and astrocytes^72^	Neurological disorders (AD^73^^,^^74^ and PD^75^)

The table shows where MPO can be localised and the disease where this enzyme participates.

MPO present in the brain originates not only from microglia but also from astrocytes and neurons. Microglia are the most commonly reported cells expressing MPO in the brain; however, MPO is aberrantly expressed in astrocytes in patients with AD^76^. It is important to mention that in healthy brains, brain microglia are in a “resting” state, and MPO is almost do not express, however in AD brains, microglia is activated and MPO expression and activity increase^10^.

**Figure 4.**
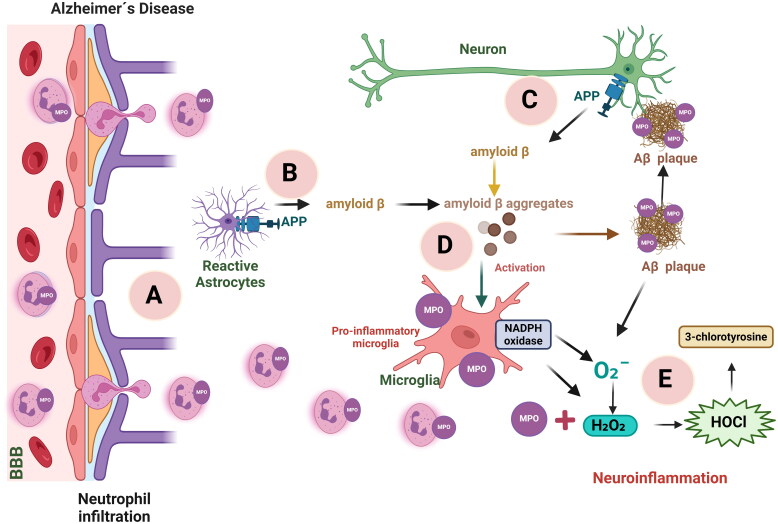
The role of myeloperoxidase (MPO) in Alzheimer’s disease. In the brain, MPO can originate from neutrophil infiltration through changes in the vasculature (A) or from astrocytes or microglia cells. Amyloid beta (Aβ) is produced by the amyloid precursor protein (APP) hydrolysis from astrocytes (B) or neurons (C). Aβ is aggregated and can activate microglial cells (D) which expressed MPO and NADPH oxidase, this produce superoxide anion (O_2_^.−^) which is converted to hydrogen peroxide (H_2_O_2_) acting as MPO substrate together chloride (Cl^−^) to form hypochlorous acid (HOCl)(E). Also, Aβ aggregates can produce H_2_O_2_ contributing to oxidative stress and neurotoxicity. Figure created using Biorender software.

In a healthy state, neutrophils flow through cerebral blood vessels; however, when an inflammatory process begins, the expression of chemokines and adhesion molecules in cerebral vessels leads to the migration of neutrophils to the brain parenchyma and therefore to increased MPO concentrations^72^^,^^73^. Neutrophils are the cells with the highest percentage of MPO protein^58^ (Figure 4).

Aβ plaque generation contributes to neuroinflammation via the activation of microglia and astrocytes. However, reactive astrocytes are also able to produce Aβ, increasing its level, and subsequently, the capacity of astrocytes and microglia to phagocytise these Aβ plaques diminishes^77^. In the context of Aβ plaques, microglia release more cytokines and recruit more microglia to plaque deposits. Upon failure to clear plaques, more cytokines are released. Peripheral macrophages may then be recruited with the aim of enhancing plaque clearance, as illustrated in Figure 4; however, the presence of these cells increases reactive oxygen species (ROS) production, resulting in neurodegeneration. The vicious cycle created by the immune response and the generation of ROS is an important physiopathological mechanism strongly implicated in AD progression^78^. However, depending on the patient’s condition, each of these mechanisms could be the first to occur. For example, neutrophil infiltration could be due to the damage initially generated by the amyloid plaques. Alternatively, if the patient has some condition that promotes blood–brain barrier damage, neutrophil infiltration generates ROS, which stimulate A production and subsequently microglia and astrocyte activation, as shown in Figure 4.

Studies in plasma from healthy individuals and AD patients have shown that MPO levels are increased in AD^1^^,^^24^. Furthermore, a correlation between MPO plasma levels and Aβ_1–40_ was verified when plasma samples from healthy elderly patients were compared with those from elderly AD patients. AD patients had elevated MPO levels (132.8 ± 114.8 ng/mL) and Aβ_1-40_ (40 ± 8 pg/mL) levels compared to healthy patients (55 ± 42.6 ng/mL and 36 ± 7 pg/mL). In addition, the Aβ_1–40_ and MPO concentrations are correlated with the disease stage, indicating that MPO represents a good biomarker for AD^24^.

In addition, interesting work has been published concerning MPO brain localisation in AD patients and control patients. The frontal cortex and hippocampus (*n* = 20–25) regions were analysed, and MPO was localised more closely to small blood vessels than to larger blood vessels or to the brain parenchyma near blood vessels. In samples from the hippocampus of AD patients, MPO was localised near amyloid plaques. Compared with those in control patients, the number of MPO-positive cells in the cortex is greater in patients with AD^15^.

In addition to the reactive species previously mentioned, MPO levels are elevated in microglia, specifically around amyloid plaques, and Aβ aggregates increase MPO mRNA expression in microglia-like cells *in vitro*^24^^,^^32^. MPO also catalyse the formation of nitrotyrosine-modified proteins and cause advanced modifications of glycation end products that are evident in AD^24^^,^^39^.

MPO regulates MMP enzymes, which are associated with neurodegenerative diseases. These enzymes favour leukocyte infiltration through the BBB, degrading the extracellular matrix of the basal lamina. *In vivo* studies in mice have shown that MPO inhibition decreases MMP activity^79^.

On the other hand, Volkman et al. used an 5XFAD mouse model of AD with MPO-deficient mice and mice with haematologic MPO deficiency (5XFAD-MPO-KO), and behavioural tests revealed significantly superior performance in spatial learning and memory, associative learning and anxiety/risk assessment behaviour compared with 5XFAD mice transplanted with WT cells (5XFAD-WT). In addition, immunohistochemical and hippocampal mRNA expression analyses revealed significantly reduced levels of inflammatory mediators in 5XFAD-MPO-KO mice, with no apparent difference in the number of Aβ plaques. APOE was reduced in 5XFAD-MPO-KO mice. The results demonstrated the relationship between neutrophil-derived MPO and the pathogenesis of the 5XFAD model of AD; therefore, MPO inhibition represents a possible therapeutic target in AD^80^.

In other studies, such as that performed by Smyth et al. in an APP/PS1 mouse model and in humans, they reported the accumulation of neutrophils in AD due to vascular changes that promote neutrophil adhesion and an increase in MPO and extracellular MPO deposits around the plaques^73^.

## HOCl as a diagnostic method for Alzheimer’s disease

Currently, AD is diagnosed post-mortem, and current diagnostic methods, which are based on behavioural and cognitive impairment or central biomarker imaging in the brain, offer poor to moderate efficacy and are not useful for early disease detection. Specific markers for the diagnosis of this disease have been proposed, including HOCl and its oxidation product 3-chlorotyrosine. MPO is the only mammalian haem enzyme capable of generating HOCl, which facilitates the identification of enzymatically active MPO in different tissues^58^. In the halogenation cycle of MPO, hypohalous acids are generated (HOX; X = Cl, Br, I, and SCN). However, MPO can use other halogens instead of Cl, such as Br, I and SCN, depending on the halogen concentration in the plasma (∼100 mM Cl^−^, 20–100 μM Br^−^, < 1 μM I^−^, 20–100 μM SCN^−^). At neutral pH values, MPO primarily generates HOCl and HOSCN ^81^.

HOCl generated by MPO catalysis is associated with neuronal cell death during neuroinflammation and the aetiology of AD. Therefore, it is important to employ effective tools for the *in vivo* detection of HOCl during the early pathology of AD^82^. AD exhibits elevated levels of HOCl, which could serve, along with MPO, as a potential biomarker for AD diagnosis.

In 2019, Samanta et al. developed switchable coumarin–morpholine conjugates for the specific detection of HOCl produced and localised with amyloid plaques using fluorescence, obtaining high selectivity and sensitivity owing to the ability of the thioamide probe to cross the BBB. This study enabled the detection, imaging, and differential quantification of HOCl in cell media and in the mouse brain^83^.

In 2022, Jia Ke et al. proposed a method for HOCl detection involving the use of a two-photon fluorogenic probe called Q-HOCl for the specific and sensitive detection of HOCl in the brains of patients with AD. The probe easily crosses the BBB, resulting in a fast response (20 s)^82^.

Coumarin boronic acid (CBA), fluorescein-based boronic acid (FLBA), 7-(p-aminophenyl)coumarin (APC), 3′-(p-aminophenyl)-fluorescein (APF), and 4-thiomorpholino-7-nitrobenz-2-oxa-1,3-diazole (NBD-TM) are small molecules that function as MPO inhibitors, and their use in diagnostic methods have been assessed^84^.

## The use of MPO inhibitors could aid in the treatment of AD

Patients with AD exhibit changes in their vasculature, which makes it easier for neutrophils to infiltrate. Although MPO is produced in microglia, astrocytes and neurons, the highest MPO concentration is produced by infiltrating neutrophils, resulting in HOCl-mediated oxidation of biomolecules in the brain; thus, the inhibition of MPO could serve as a therapeutic target in AD patients focused on reducing cellular and cognitive damage^72^^,^^73^.

MPO inhibitors can act in different ways: as reversible or irreversible inhibitors and by promoting the accumulation of compound II^85^^,^^86^.

Reversible inhibitors are molecules with high affinity for the MPO active site or with its haem group via noncovalent interactions. Even in the inactive form of the enzyme, some reversible inhibitors maintain their interactions with the active site to prevent reduction by other substrates and reactivation of the native enzyme^86^^,^^87^.

In contrast, irreversible inhibitors are molecules with a high affinity for the haem iron atom forming covalently bond, inactivating or destroying the haem group^86^^,^^87^. Recently, AZD4831, an irreversible MPO inhibitor, was reported as a potential treatment for heart failure, and this compound has been evaluated in clinical trials^86^^,^^88^.

In addition, other reversible inhibitors act via the accumulation of compound II. These compounds are small molecules that are easily oxidised by MPO or form a MPO-complex preventing the continuation of MPO peroxidation cycle which allow the formation and accumulation of compound II^89^. However, many molecules *in vivo* can reduce compound II returning MPO to its native form^85^^,^^87^. In addition, MPO activity can be inhibit by the H_2_O_2_ scavenging enzymes catalase and glutathione peroxidase or well the oxidative damage produced by HOCl can be prevented using HOCl scavenger such as chlorogenic acid^90^.

Several natural compounds with antioxidant activity, such as polyphenols, have inhibitory effects on MPO. For example, quercetin inhibits MPO activity and inhibits the oxidation of low-density lipoprotein (LDL) mediated by neutrophils; however, other compounds from natural resources that inhibit MPO, such as resveratrol, ferulic and caffeic acids, exhibit low inhibitory activity^91^.

Furthermore, organic acids naturally abundant in plants has been evaluated as MPO inhibitors on its peroxidation, chlorination, and nitration activities showed that oxalic acid was the acid with the best MPO-inhibition in relation to fumaric, acetic and succinic acids^92^. In addition, phenolic compounds were extract from *Populus nigra* employing lactic acid as solvent during the extraction. The MPO inhibitory activity of the extract was evaluated having a IC50 of 0.084%, identifying that the compounds esculoside and rhamnosyl-hexosyl-acyl-quercetin were the principally compounds responsible of MPO inhibition^93^.

In addition, nonsteroidal anti-inflammatory drugs (NSAIDs) have been evaluated as MPO inhibitors, and several of these drugs have reach clinical trials for AD. Ibuprofen has been evaluated to determine its effect on A levels and has also been employed in combination with cromolyn in clinical trials, starting in 2021. The drugs assessed included ALZT-OP1 (cromolyn and ibuprofen), ALZT-OP1a (cromolyn) and ALZT-OP1b (ibuprofen) (ClinicalTrials ID: NCT04570644; NCT00239746). Although ibuprofen is one of the most studied NSAIDs, insufficient results are available; however, has been demonstrated that ibuprofen is a better COX2 inhibitor than an MPO inhibitor.

Another NSAID is aspirin, which, although not a specific MPO inhibitor, may reduce MPO activity indirectly by inhibiting inflammation and microglial activation^94^^,^^95^.

AZD3241, a selective and irreversible inhibitor of myeloperoxidase (MPO), is currently being assessed in clinical trials. Research has suggested that AZD3241 reduces oxidative stress and chronic neuroinflammation, which are significant factors in neurodegenerative diseases. In patients with Parkinson’s disease, MPO inhibition by AZD3241 significantly affects microglial activity. These findings support further studies on the efficacy of AZD3241 in the treatment of neurodegenerative disorders^96^. In this sense, AstraZeneca has focused considerable attention on obtaining MPO inhibitors to treat different pathologies, with the aim of reducing the damage caused by HOCl.

Some MPO inhibitors or their derivatives have also been shown to be effective in the treatment of AD, acting not only on MPO but also on enzymes involved in the cholinergic pathway, such as acetylcholinesterase (AChE) and butyrylcholinesterase (BChE), as well as on histone deacetylase 6 (HDAC6), which has dual activity, as shown in Table 2.

**Table 2.** Myeloperoxidase (MPO) inhibitors, acting also on targets involved in AD.

MPO inhibitor	Type of inhibition	Target in AD
Tryptamine derivatives^83^	Reversible	AChE inhibitor^16^^,^^97^; BChE inhibitor^16^^,^^17^; neuroprotective, AO and antineuroinflammatory properties^97^^,^^16^^,^^17^.
Hydroxamic acid^10^	Reversible	HDAC6 inhibitor^98^, anti-aggregation properties against Aβ peptides, neuroprotective properties^99^.
4-aminobenzoic acid hydrazides^100^	Irreversible	Neurogenesis^101^
Lansoprazole^102^	–	Neuroprotective, AChE inhibitor, AO, anti-inflammatory properties^102^.
Rivastigmine^14^	–	AChE inhibition^14^.

The type of MPO inhibition is mentioned.

**Figure 5.**
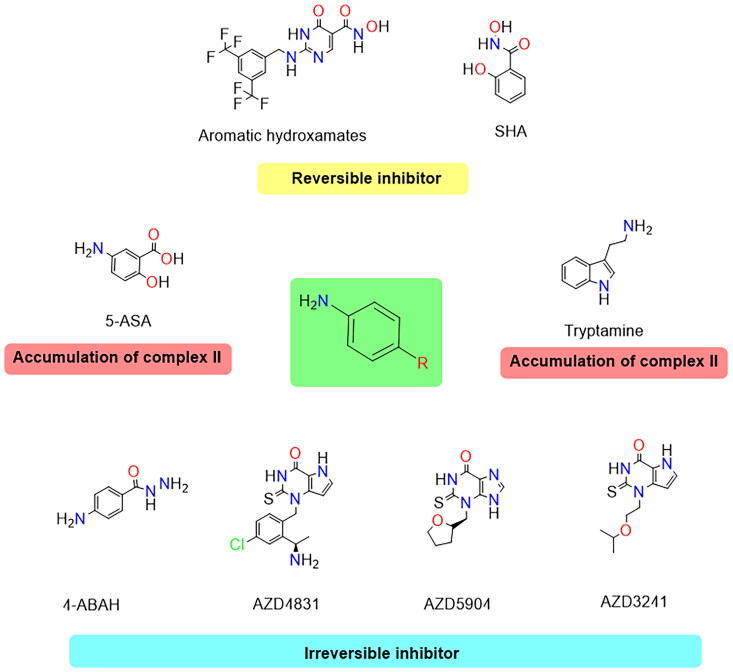
Chemical structures of myeloperoxidase inhibitors. The chemical structures of the aromatic hydroxamates SHA, 5-ASA, tryptamine, 4-ABAH, AZD4831, AZD5904 and AZD3241 are shown. These compounds demonstrate different mechanisms of action against MPO (reversible inhibition, accumulation of compound II and irreversible inhibition). Figure created using Biorender software.

Figure 5 shows several chemical structures of the MPO inhibitors, all of which feature an aromatic ring and primary, secondary, and tertiary amines attached to it. The presence of these aromatic rings and amino groups allows the compounds to interact with the enzyme’s active site, stabilising its inactive form or preventing the formation of reactive intermediates.

Recently has been reported compounds that have amide, hydrazide and hydroxamic acid groups either on nitrogen or on Sulphur atom being the compound 2–(2-thioxo-2,3-dihydro-1H-benzo[d]imidazole-1-yl)acetohydrazide (C19) the most promissory having MPO-inhibitory activity on the chlorination and peroxidation cycles^103^.

Derivates of pyrrolidinone indole (IN-4) has been also evaluated as selective MPO inhibitor over thyroid peroxidase (TPO), and on extracellular MPO over intracellular MPO. After dialysis study the MPO activity was evaluated post-incubation of MPO with IN-4, acting this compound as a possible irreversible MPO inhibitor^104^.

7-chloro-quinolines as been also evaluated as MPO inhibitors by in silico studies where was found that by a molecular dynamic simulations the best compound named 14 maintain its interactions in the catalytic sites suggesting a stability of the complex formed between MPO and the compound 14^105^.

Various MPO inhibitors have shown effects on AD, such as tryptamine derivatives that act as reversible MPO inhibitors (5-fluorotryptamine and 5-chlorotryptamine with IC_50_ values of 0.79 mM and 0.73 mM, respectively)^106^. These compounds also inhibit AChE with IC_50_ > 100 µM and BChE with IC_50_ = 7 nM, exhibiting neuroprotective, antioxidant and anti-inflammatory activity. One example of such an inhibitor includes (3–(2-(2-(phenylamino)benzamido)ethyl)-1*H*-indol-5-yl heptylcarbamate (6H6))^97^.

Another molecule studied for both MPO inhibition and AD is hydroxymic acid, which reversibly inhibits MPO (salicylhydroxamic acid (SHA) IC_50_ = 25 µM)^18^. Additionally, because it can chelate metals such as Fe and Zn, it is a good inhibitor of enzymes such as HDACs^98^.

At a concentration of 10 µmol/L, rivastigmine inhibits MPO activity by 17.1 ± 11.4%. Rivastigmine inhibits AChE with an IC_50_ > 1535 nM and BChE with an IC_50_ = 301 nM^14^^,^^17^.

Based on this proposal, in 2020, Correa-Santos et al. evaluated acylhydrazone compounds derived from isoniazid as antioxidants and inhibitors of MPO and AChE and reported that one of the compounds showed both antioxidant and inhibitory effects on MPO (80 ± 9%) and AChE (54 ± 2%) *in vitro* at a concentration of 100 µM. *In silico,* these compounds interacted with the active site of the enzymes, where the aromatic ring plays an important role in inhibition^14^.

These studies showed that MPO can be an important target for the design of drugs against AD and that this approach can have a multitarget effect not only on MPO but also on other targets involved in AD. Then, more studies could be conducted to evaluate some drugs used in AD that present inhibitory activity against β-secretase, gamma-secretase, tumour necrosis factor alpha (TNF-ɑ) and interleukin-6 (IL-6), such as diosgenin and pterostilbene^107^.

## Conclusions

Myeloperoxidase (MPO) plays a role in the development of neurodegenerative diseases such as Alzheimer’s disease. A detailed understanding of the precise mechanisms of MPO in the brain and its interaction with the amyloid beta peptide is crucial and requires further exploration. Understanding these mechanisms is essential for the development of drugs specifically targeting MPO in AD. Multitarget molecules that, in addition to being MPO inhibitors, can inhibit Aβ aggregation have been identified. Some of these specific MPO inhibitors have advanced to clinical trials based on their promising preclinical results. MPO inhibitors are not currently marketed; thus, the development of new inhibitors as investigational drugs is a significant and promising field of research.

## Data Availability

Data sharing is not applicable to this article, as no new data were created or analysed in this study.

## References

[1] Bawa KK, Krance SH, Herrmann N, Cogo-Moreira H, Ouk M, Yu D, Wu C-Y, Black SE, Lanctôt KL, Swardfager W. A peripheral neutrophil-related inflammatory factor predicts a decline in executive function in mild Alzheimer’s disease. J Neuroinflammation. 2020;17(1):84. 32171317 10.1186/s12974-020-01750-3 PMC7071641

[2] Swomley AM, Förster S, Keeney JT, Triplett J, Zhang Z, Sultana R, Butterfield DA. Abeta, oxidative stress in Alzheimer disease: evidence based on proteomics studies. Biochim Biophys Acta. 2014;1842(8):1248–1257. 24120836 10.1016/j.bbadis.2013.09.015 PMC3981962

[3] Andrés CMC, Pérez de la Lastra JM, Juan CA, Plou FJ, Pérez-Lebeña E. Hypochlorous acid chemistry in mammalian cells—influence on infection and role in various pathologies. Int J Mol Sci. 2022;23(18):10735. 36142645 10.3390/ijms231810735 PMC9504810

[4] Pravalika K, Sarmah D, Kaur H, Wanve M, Saraf J, Kalia K, Borah A, Yavagal DR, Dave KR, Bhattacharya P, et al. Myeloperoxidase and neurological disorder: a crosstalk. ACS Chem Neurosci. 2018;9(3):421–430. 29351721 10.1021/acschemneuro.7b00462

[5] Whiteman M, Cheung NS, Zhu Y-Z, Chu SH, Siau JL, Wong BS, Armstrong JS, Moore PK. Hydrogen sulphide: a novel inhibitor of hypochlorous acid-mediated oxidative damage in the brain? Biochem Biophys Res Commun. 2005;326(4):794–798. 15607739 10.1016/j.bbrc.2004.11.110

[6] Yap YW, Whiteman M, Bay BH, Li Y, Sheu F-S, Qi RZ, Tan CH, Cheung NS. Hypochlorous acid induces apoptosis of cultured cortical neurons through activation of calpains and rupture of lysosomes. J Neurochem. 2006;98(5):1597–1609. 16923169 10.1111/j.1471-4159.2006.03996.x

[7] Tidén A-K, Sjögren T, Svensson M, Bernlind A, Senthilmohan R, Auchère F, Norman H, Markgren P-O, Gustavsson S, Schmidt S, et al. 2-Thioxanthines are mechanism-based inactivators of myeloperoxidase that block oxidative stress during inflammation. J Biol Chem. 2011;286(43):37578–37589. 21880720 10.1074/jbc.M111.266981 PMC3199503

[8] Nicholls SJ, Hazen SL. Myeloperoxidase and cardiovascular disease. Arterioscler Thromb Vasc Biol. 2005;25(6):1102–1111. 10.1161/01.ATV.0000163262.83456.6d 15790935

[9] Frangie C, Daher J. Role of myeloperoxidase in inflammatioand atherosclerosis. Biomed Rep. 2022;16(6):53. 35620311 10.3892/br.2022.1536 PMC9112398

[10] Lin W, Chen H, Chen X, Guo C. The roles of neutrophil-derived Myeloperoxidase (MPO) in diseases: the new progress. Antioxidants (Basel). 2024;13(1):132. 38275657 10.3390/antiox13010132 PMC10812636

[11] Aries ML, Hensley-Mcbain T. Neutrophils as a potential therapeutic target in Alzheimer’s Disease. Front Immunol*.* 2023;14:1123149. 10.3389/fimmu.2023.1123149 PMC10020508 36936930

[12] Wu C, Bawa KK, Ouk M, Leung N, Yu D, Lanctôt KL, Herrmann N, Pakosh M, Swardfager W. Neutrophil activation in Alzheimer’s disease and mild cognitive impairment: a systematic review and meta-analysis of protein markers in blood and cerebrospinal fluid. Ageing Res Rev. 2020;62:101130. 10.1016/j.arr.2020.101130 32712109

[13] Ighodaro OM, Akinloye OA. First line defence antioxidants-superoxide dismutase (SOD), catalase (CAT) and glutathione peroxidase (GPX): their fundamental role in the entire antioxidant defence grid. Alexandria J Med. 2018;54(4):287–293.

[14] Santos DC, Henriques RR, Junior MAdAL, Farias AB, Nogueira TLdC, Quimas JVF, Romeiro NC, Silva LLd, Souza ALFd. Acylhydrazones as isoniazid derivatives with multi-target profiles for the treatment of Alzheimer’s disease: radical scavenging, myeloperoxidase/acetylcholinesterase inhibition and biometal chelation. Bioorg Med Chem. 2020;28(10):115470. 32278712 10.1016/j.bmc.2020.115470

[15] Gellhaar S, Sunnemark D, Eriksson H, Olson L, Galter D. Myeloperoxidase-immunoreactive cells are significantly increased in brain areas affected by neurodegeneration in Parkinson’s and Alzheimer’s disease. Cell Tissue Res. 2017;369(3):445–454. 28466093 10.1007/s00441-017-2626-8 PMC5579172

[16] Liu D, Zhang H, Wang Y, Liu W, Yin G, Wang D, Li J, Shi T, Wang Z. Design, synthesis, and biological evaluation of carbamate derivatives of N-salicyloyl tryptamine as multifunctional agents for the treatment of Alzheimer’s disease. Eur J Med Chem. 2022;229:114044. 34923430 10.1016/j.ejmech.2021.114044

[17] Wang Y, Zhang H, Liu D, Li X, Long L, Peng Y, Qi F, Wang Y, Jiang W, Wang Z, et al. Discovery of carbamate-based N-salicyloyl tryptamine derivatives as novel pleiotropic agents for the treatment of Alzheimer’s disease. Bioorg Chem. 2022;127(March):105993. 35834980 10.1016/j.bioorg.2022.105993

[18] Forbes LV, Sjögren T, Auchère F, Jenkins DW, Thong B, Laughton D, Hemsley P, Pairaudeau G, Turner R, Eriksson H, et al. Potent reversible inhibition of myeloperoxidase by aromatic hydroxamates. J Biol Chem. 2013;288(51):36636–36647. 24194519 10.1074/jbc.M113.507756 PMC3868775

[19] Zámocký M, Hofbauer S, Schaffner I, Gasselhuber B, Nicolussi A, Soudi M, Pirker KF, Furtmüller PG, Obinger C. Independent evolution of four heme peroxidase superfamilies. Arch Biochem Biophys. 2015;574:108–119. 25575902 10.1016/j.abb.2014.12.025 PMC4420034

[20] Koua D, Cerutti L, Falquet L, Sigrist CJA, Theiler G, Hulo N, Dunand C. PeroxiBase: A database with new tools for peroxidase family classification. Nucleic Acids Res. 2009;37(Database issue):D261–D266. 18948296 10.1093/nar/gkn680 PMC2686439

[21] Khan AA, Rahmani AH, Aldebasi YH, Aly SM. Biochemical and pathological studies on peroxidases -an updated review. Glob J Health Sci. 2014;6(5):87–98. 25168993 10.5539/gjhs.v6n5p87 PMC4825458

[22] Vlasova II. Peroxidase activity of human hemoproteins: keeping the fire under control. Molecules. 2018;23(10):2561. 30297621 10.3390/molecules23102561 PMC6222727

[23] Arnhold J, Malle E. Halogenation activity of mammalian heme peroxidases. Antioxidants (Basel). 2022;11(5):890. 35624754 10.3390/antiox11050890 PMC9138014

[24] Tzikas S, Schlak D, Sopova K, Gatsiou A, Stakos D, Stamatelopoulos K, Stellos K, Laske C. Increased myeloperoxidase plasma levels in patients with alzheimer’s disease. J Alzheimers Dis. 2014;39(3):557–564. 24217274 10.3233/JAD-131469

[25] Li Y, Ganesh T, Diebold BA, Zhu Y, McCoy JW, Smith SME, Sun A, Lambeth JD. Thioxo-dihydroquinazolin-one compounds as novel inhibitors of myeloperoxidase. ACS Med Chem Lett. 2015;6(10):1047–1052. 26487910 10.1021/acsmedchemlett.5b00287 PMC4601060

[26] Arnhold J. The dual role of myeloperoxidase in immune response. Int J Mol Sci. 2020;21(21):8057. 33137905 10.3390/ijms21218057 PMC7663354

[27] Vanhamme L, Boudjeltia KZ, Antwerpen PV, Delporte C. The other myeloperoxidase: Emerging functions. Arch Biochem Biophys. 2018;649(December 2017):1–14. 29614255 10.1016/j.abb.2018.03.037

[28] Ikeda-Saito M. On the analogy in the structure of the spleen green heme protein and granulocyte myeloperoxidase. FEBS Lett. 1986;202(2):245–250. 3013687 10.1016/0014-5793(86)80695-0

[29] Reiding KR, Franc XV, Huitema MG, Brouwer E, Heeringa P, Heck XAJR. Neutrophil myeloperoxidase harbors distinct site-specific peculiarities in its glycosylation. J Biol Chem. 2019;294(52):20233–20245. 31719144 10.1074/jbc.RA119.011098 PMC6937560

[30] Agner K. Verdoperoxidase: a ferment isolated from leukocytes. Acta Physiol Scand. 1943;3:137–148.

[31] Teng N, Maghzal GJ, Talib J, Rashid I, Lau AK, Stocker R. The roles of myeloperoxidase in coronary artery disease and its potential implication in plaque rupture. Redox Rep. 2017;22(2):51–73. 27884085 10.1080/13510002.2016.1256119 PMC6837458

[32] Green PS, Mendez AJ, Jacob JS, Crowley JR, Growdon W, Hyman BT, Heinecke JW. Neuronal expression of myeloperoxidase is increased in Alzheimer’s disease. J Neurochem. 2004;90(3):724–733. 15255951 10.1111/j.1471-4159.2004.02527.x

[33] Davies MJ, Hawkins CL. The role of myeloperoxidase in biomolecule modification, chronic inflammation, and disease. Antioxid Redox Signal. 2020;32(13):957–981. 31989833 10.1089/ars.2020.8030

[34] Wang H-P, Wu H-Y, Ma C-L, Zeng Q-T, Zhu K-M, Cui S-M, Li H-L, Wu G-T, Wu Z-W, He J-Z, et al. Optimal formula of *Angelica sinensis* ameliorates memory deficits in β-amyloid protein-induced Alzheimer’s disease rat model. Curr Med Sci. 2022;42(1):39–47. 35122611 10.1007/s11596-022-2528-1

[35] Huang WJ, Zhang X, Chen WW. Role of oxidative stress in Alzheimer’s disease. Biomed Rep. 2016;4(5):519–522. 27123241 10.3892/br.2016.630 PMC4840676

[36] Hornemann T. Mini review: lipids in peripheral nerve disorders. Neurosci Lett. 2021;740:135455. 33166639 10.1016/j.neulet.2020.135455

[37] Naudí A, Cabré R, Jové M, Ayala V, Gonzalo H, Portero-Otín M, Ferrer I, Pamplona R. Lipidomics of human brain aging and Alzheimer’s disease pathology. Int Rev Neurobiol. 2015;122:133–189. 10.1016/bs.irn.2015.05.008 26358893

[38] Vinokurov AY, Stelmashuk OA, Ukolova PA, Zherebtsov EA, Abramov AY. Brain region specificity in reactive oxygen species production and maintenance of redox balance. Free Radic Biol Med. 2021;174(August):195–201. 34400296 10.1016/j.freeradbiomed.2021.08.014

[39] Su B, Wang X, Nunomura A, Moreira PI, Lee H-g, Perry G, Smith MA, Zhu X. Oxidative stress signaling in Alzheimers disease. Curr Alzheimer Res. 2008;5(6):525–532. 19075578 10.2174/156720508786898451 PMC2780015

[40] Smith DG, Cappai R, Barnham KJ. The redox chemistry of the Alzheimer’s disease amyloid β peptide. Biochim Biophys Acta. 2007;1768(8):1976–1990. 17433250 10.1016/j.bbamem.2007.02.002

[41] Cassidy L, Fernandez F, Johnson JB, Naiker M, Owoola AG, Broszczak DA. Oxidative stress in Alzheimer’s disease: a review on emergent natural polyphenolic therapeutics. Complement Ther Med. 2020;49(vember 2019):102294. 32147039 10.1016/j.ctim.2019.102294

[42] Skoumalová A, Hort J. Blood markers of oxidative stress in Alzheimer’s disease. J Cell Mol Med. 2012;16(10):2291–2300. 22564475 10.1111/j.1582-4934.2012.01585.x PMC3823422

[43] Yang WN, Han H, Hu XD, Feng GF, Qian YH. The effects of perindopril on cognitive impairment induced by d-galactose and aluminum trichloride via inhibition of acetylcholinesterase activity and oxidative stress. Pharmacol Biochem Behav. 2013;114–115:31–36. 10.1016/j.pbb.2013.10.027 24201055

[44] Schröter J, Schiller J. Chlorinated phospholipids and fatty acids: (Patho)physiological relevance, potential toxicity, and analysis of lipid chlorohydrins. Oxid Med Cell Longev. 2016;2016(1):8386362. 28090245 10.1155/2016/8386362 PMC5206476

[45] Hawkins CL, Pattison DI, Davies MJ. Reaction of protein chloramines with DNA and nucleosides: evidence for the formation of radicals, protein-DNA cross-links and DNA fragmentation. Biochem J. 2002;365(Pt 3):605–615. 12010123 10.1042/BJ20020363 PMC1222737

[46] Krewing M, Stepanek JJ, Cremers C, Lackmann J-W, Schubert B, Müller A, Awakowicz P, Leichert LIO, Jakob U, Bandow JE. The molecular chaperone Hsp33 is activated by atmospheric-pressure plasma protecting proteins from aggregation. J R Soc Interface. 2019;16(155):20180966. 31213177 10.1098/rsif.2018.0966 PMC6597770

[47] Beavers WN, Skaar EP. Neutrophil-generated oxidative stress and protein damage in *Staphylococcus aureus*. Pathog Dis. 2016;74(6):ftw060. 27354296 10.1093/femspd/ftw060 PMC5975594

[48] Ashoka AH, Ali F, Tiwari R, Kumari R, Pramanik SK, Das A. Recent Advances in Fluorescent Probes for Detection of HOCl and HNO. ACS Omega. 2020;5(4):1730–1742. 32039308 10.1021/acsomega.9b03420 PMC7003195

[49] Goemans CV, Collet JF. Stress-induced chaperones: a first line of defense against the powerful oxidant hypochlorous acid [version 1; peer review: 4 approved]. F1000Res. 2019;8:1678. 10.12688/f1000research.19517.1 PMC6758839 31583082

[50] Jeitner TM, Kalogiannis M, Patrick PA, Gomolin I, Palaia T, Ragolia L, Brand D, Delikatny EJ. Inflaming the diseased brain: a role for tainted melanins. Biochim Biophys Acta. 2015;1852(5):937–950. 25585261 10.1016/j.bbadis.2015.01.004 PMC5113040

[51] Rayner BS, Love DT, Hawkins CL. Comparative reactivity of myeloperoxidase-derived oxidants with mammalian cells. Free Radic Biol Med. 2014;71:240–255. 24632382 10.1016/j.freeradbiomed.2014.03.004

[52] Winterbourn CC, Brennan SO. Characterization of the oxidation products of the reaction between reduced glutathione and hypochlorous acid. Biochem J. 1997;326 (Pt 1)(Pt 1):87–92. 9337854 10.1042/bj3260087 PMC1218640

[53] Kearns S, Dawson R. Cytoprotective effect of taurine against hypochlorous acid toxicity to PC12 cells. Adv Exp Med Biol. 2000;483:563–570. 11787641 10.1007/0-306-46838-7_60

[54] Yap YW, Whiteman M, Cheung NS. Chlorinative stress: an under appreciated mediator of neurodegeneration? Cell Signal. 2007;19(2):219–228. 16959471 10.1016/j.cellsig.2006.06.013

[55] Wang Y, Chuang CY, Hawkins CL, Davies MJ. Activation and inhibition of Human Matrix Metalloproteinase-9 (MMP9) by HOCl, myeloperoxidase and chloramines. Antioxidants (Basel). 2022;11(8):1616. 10.3390/antiox11081616 PMC9405048 36009335

[56] Schraufstätter IU, Browne K, Harris A, Hyslop PA, Jackson JH, Quehenberger O, Cochrane CG. Mechanisms of hypochlorite injury of target cells. J Clin Invest. 1990;85(2):554–562. 2153710 10.1172/JCI114472 PMC296458

[57] Ray RS, Katyal A. Myeloperoxidase: bridging the gap in neurodegeneration. Neurosci Biobehav Rev. 2016;68:611–620. 27343997 10.1016/j.neubiorev.2016.06.031

[58] Ulfig A, Leichert LI. The effects of neutrophil-generated hypochlorous acid and other hypohalous acids on host and pathogens. Cell Mol Life Sci. 2021;78(2):385–414. 32661559 10.1007/s00018-020-03591-y PMC7873122

[59] Brown KE, Brunt EM, Heinecke JW. Immunohistochemical detection of myeloperoxidase and its oxidation products in Kupffer cells of human liver. Am J Pathol. 2001;159(6):2081–2088. 11733358 10.1016/S0002-9440(10)63059-3 PMC1850615

[60] Zhu A, Ge D, Zhang J, Teng Y, Yuan C, Huang M, Adcock IM, Barnes PJ, Yao X. Sputum myeloperoxidase in chronic obstructive pulmonary disease. Eur J Med Res. 2014;19(1):12. 24588870 10.1186/2047-783X-19-12 PMC4016613

[61] Bobryshev YV, Ivanova EA, Chistiakov DA, Nikiforov NG, Orekhov AN. Macrophages and their role in atherosclerosis: pathophysiology and transcriptome analysis. Biomed Res Int. 2016;2016(Figure 1):9582430–9582413. 27493969 10.1155/2016/9582430 PMC4967433

[62] Muzio L, Viotti A, Martino G. Microglia in neuroinflammation and neurodegeneration: from understanding to therapy. Front Neurosci. 2021;15(September):742065. 34630027 10.3389/fnins.2021.742065 PMC8497816

[63] Spangenberg EE, Green KN. Inflammation in Alzheimer’s disease: lessons learned from microglia-depletion models. Brain Behav Immun. 2017;61:1–11. 27395435 10.1016/j.bbi.2016.07.003 PMC5218993

[64] Sugiyama S, Okada Y, Sukhova GK, Virmani R, Heinecke JW, Libby P. Macrophage myeloperoxidase regulation by granulocyte macrophage colony-stimulating factor in human atherosclerosis and implications in acute coronary syndromes. Am J Pathol. 2001;158(3):879–891. 11238037 10.1016/S0002-9440(10)64036-9 PMC1850342

[65] Breckwoldt MO, Chen JW, Stangenberg L, Aikawa E, Rodriguez E, Qiu S, Moskowitz MA, Weissleder R. Tracking the inflammatory response in stroke in vivo by sensing the enzyme myeloperoxidase. Proc Natl Acad Sci U S A. 2008;105(47):18584–18589. 19011099 10.1073/pnas.0803945105 PMC2587593

[66] Siraki AG. The many roles of myeloperoxidase: from inflammation and immunity to biomarkers, drug metabolism and drug discovery. Redox Biol. 2021;46:102109. 34455146 10.1016/j.redox.2021.102109 PMC8403760

[67] Koop AC, Thiele ND, Steins D, Michaëlsson E, Wehmeyer M, Scheja L, Steglich B, Huber S, Schulze Zur Wiesch J, Lohse AW, et al. Therapeutic targeting of myeloperoxidase attenuates NASH in mice. Hepatol Commun. 2020;4(10):1441–1458. 33024915 10.1002/hep4.1566 PMC7527691

[68] Pileri SA, Facchetti F, Ascani S, Sabattini E, Poggi S, Piccioli M, Rondelli D, Vergoni F, Zinzani PL, Piccaluga PP, et al. Myeloperoxidase expression by histiocytes in Kikuchi’s and Kikuchi-like lymphadenopathy. Am J Pathol. 2001;159(3):915–924. 11549584 10.1016/S0002-9440(10)61767-1 PMC1850446

[69] Jang SJ, Min Jeon H, Kim D, Yang WI. Myeloperoxidase positive histiocytes in subacute necrotizing lymphadenitis express both CD11c and CD163. Basic Appl Pathol. 2011;4(4):110–115.

[70] Souparnika S, D’Souza B, D’Souza V, Kumar S, Manjrekar P, Bairy M, Parthasarathy R, Kosuru S. Emerging role of myeloperoxidase in the prognosis of nephrotic syndrome patients before and after steroid therapy. J Clin Diagn Res. 2015;9(7):BC01–4. 10.7860/JCDR/2015/12532.6222 PMC4572946 26393116

[71] Zhao X, Lin S, Li H, Si S, Wang Z. Myeloperoxidase controls bone turnover by suppressing osteoclast differentiation through modulating reactive oxygen species level. J Bone Miner Res. 2021;36(3):591–603. 33289180 10.1002/jbmr.4215 PMC7988577

[72] Chen S, Chen H, Du Q, Shen J. Targeting Myeloperoxidase (MPO) mediated oxidative stress and inflammation for reducing brain ischemia injury: potential application of natural compounds. Front Physiol. 2020;11(May):433. 32508671 10.3389/fphys.2020.00433 PMC7248223

[73] Smyth LCD, Murray HC, Hill M, van Leeuwen E, Highet B, Magon NJ, Osanlouy M, Mathiesen SN, Mockett B, Singh-Bains MK, et al. Neutrophil-vascular interactions drive myeloperoxidase accumulation in the brain in Alzheimer’s disease. Acta Neuropathol Commun. 2022;10(1):38. 35331340 10.1186/s40478-022-01347-2 PMC8944147

[74] Davies MJ, Hawkins CL, Pattison DI, Rees MD. Mammalian heme peroxidases: from molecular mechanisms to health implications. Antioxid Redox Signal. 2008;10(7):1199–1234. 18331199 10.1089/ars.2007.1927

[75] Teismann P. Neurodegenerativer Prozess bei Morbus Parkinson: Rolle der Myeloperoxidase. Dtsch Med Wochenschr. 2013;139(03):99–102. 24277448 10.1055/s-0033-1359907

[76] Maki RA, Tyurin VA, Lyon RC, Hamilton RL, DeKosky ST, Kagan VE, Reynolds WF. Aberrant expression of myeloperoxidase in astrocytes promotes phospholipid oxidation and memory deficits in a mouse model of Alzheimer disease. J Biol Chem. 2009;284(5):3158–3169. 19059911 10.1074/jbc.M807731200 PMC2631957

[77] Frost GR, Li YM. The role of astrocytes in amyloid production and Alzheimer’s disease. Open Biol. 2017;7(12):170228. 29237809 10.1098/rsob.170228 PMC5746550

[78] Sayed A, Bahbah EI, Kamel S, Barreto GE, Ashraf GM, Elfil M. The neutrophil-to-lymphocyte ratio in Alzheimer’s disease: current understanding and potential applications. J Neuroimmunol. 2020;349(August):577398. 32977249 10.1016/j.jneuroim.2020.577398

[79] Zhang Y, Dong H, Seeburg DP, Wojtkiewicz GR, Waterman P, Pulli B, Forghani R, Ali M, Iwamoto Y, Swirski FK, et al. Multimodal molecular imaging demonstrates myeloperoxidase regulation of matrix metalloproteinase activity in neuroinflammation. Mol Neurobiol. 2019;56(2):954–962. 29808380 10.1007/s12035-018-1137-2 PMC6261713

[80] Volkman R, Ben-Zur T, Kahana A, Garty BZ, Offen D. myeloperoxidase deficiency inhibits cognitive decline in the 5XFAD mouse model of Alzheimer’s disease. Front Neurosci. 2019;13(September):990. 31611761 10.3389/fnins.2019.00990 PMC6769081

[81] Hawkins CL, Davies MJ. Role of myeloperoxidase and oxidant formation in the extracellular environment in inflammation-induced tissue damage. Free Radic Biol Med. 2021;172(June):633–651. 34246778 10.1016/j.freeradbiomed.2021.07.007

[82] Ke J, Zhao P, Li J, Fu Q. Visualization of HOCl in the brains of Alzheimer’s disease models using an easily available two-photon fluorogenic probe. J Mater Chem B. 2022;10(42):8744–8749. 36254770 10.1039/d2tb01502a

[83] Samanta S, Govindaraju T. Unambiguous detection of elevated levels of hypochlorous acid in double transgenic AD Mouse brain. ACS Chem Neurosci. 2019;10(12):4847–4853. 31790189 10.1021/acschemneuro.9b00554

[84] Pierzchała K, Pięta M, Rola M, Świerczyńska M, Artelska A, Dębowska K, Podsiadły R, Pięta J, Zielonka J, Sikora A, et al. Fluorescent probes for monitoring myeloperoxidase-derived hypochlorous acid: a comparative study. Sci Rep. 2022;12(1):9314. 35660769 10.1038/s41598-022-13317-8 PMC9166712

[85] Soubhye J, Van Antwerpen P, Dufrasne F. A patent review of myeloperoxidase inhibitors for treating chronic inflammatory syndromes (focus on cardiovascular diseases, 2013-2019). Expert Opin Ther Pat. 2020;30(8):595–608. 32510253 10.1080/13543776.2020.1780210

[86] Galijasevic S. The development of myeloperoxidase inhibitors. Bioorg Med Chem Lett. 2019;29(1):1–7. 30466896 10.1016/j.bmcl.2018.11.031

[87] Soubhye J, Furtmüller PG, Dufrasne F, Obinger C. Inhibition of Myeloperoxidase. Handb Exp Pharmacol. 2021;264:261–285. 33372235 10.1007/164_2020_388

[88] Inghardt T, Antonsson T, Ericsson C, Hovdal D, Johannesson P, Johansson C, Jurva U, Kajanus J, Kull B, Michaëlsson E, et al. Discovery of AZD4831, a mechanism-based irreversible inhibitor of myeloperoxidase, as a potential treatment for heart failure with preserved ejection fraction. J Med Chem. 2022;65(17):11485–11496. 36005476 10.1021/acs.jmedchem.1c02141 PMC9469207

[89] Soubhye J, Meyer F, Furtmüller P, Obinger C, Dufrasne F, Antwerpen PV. Characterization of chemical features of potent myeloperoxidase inhibitors. Future Med Chem. 2016;8(11):1163–1177. 27402298 10.4155/fmc-2016-0031

[90] Kono Y, Shibata H, Kodama Y, Ueda A, Sawa Y. Chlorogenic acid as a natural scavenger for hypochlorous acid. Biochem Biophys Res Commun. 1995;217(3):972–978. 8554623 10.1006/bbrc.1995.2865

[91] Tangeten C, Boudjeltia KZ, Delporte C, Van Antwerpen P, Korpak K. Unexpected role of MPO-oxidized LDLs in atherosclerosis: in between inflammation and its resolution. Antioxidants (Basel). 2022;11(5):874. 35624738 10.3390/antiox11050874 PMC9137493

[92] Sarkarati B. Inhibitory e ff ect of organic acids on human neutrophil myeloperoxidase’s peroxidation, chlorination, and nitration activities. Turkish J Biochem. 2023;48(5):485–491.

[93] Zaidi S, Benaida N, Sara D, Tebbi O, Kadi R, Saidene N. Optimization of ultrasound ‑ assisted extraction of phenolic compounds from *Populus nigra* as potential myeloperoxidase inhibitors. Chem Pap. 2024;78(5):2841–2854.

[94] Kata D, Földesi I, Feher LZ, Hackler L, Puskas LG, Gulya K. A novel pleiotropic effect of aspirin: beneficial regulation of pro- and anti-inflammatory mechanisms in microglial cells. Brain Res Bull. 2017;132:61–74. 28528204 10.1016/j.brainresbull.2017.05.009

[95] Gąsowska-Bajger B, Sosnowska K, Gąsowska-Bodnar A, Bodnar L. The effect of acetylsalicylic acid, as a representative non-steroidal anti-inflammatory drug, on the activity of myeloperoxidase. Pharmaceuticals (Basel). 2023;16(7):1012. 37513924 10.3390/ph16071012 PMC10386752

[96] Jucaite A, Svenningsson P, Rinne JO, Cselényi Z, Varnäs K, Johnström P, Amini N, Kirjavainen A, Helin S, Minkwitz M, et al. Effect of the myeloperoxidase inhibitor AZD3241 on microglia: a PET study in Parkinson’s disease. Brain. 2015;138(Pt 9):2687–2700. 26137956 10.1093/brain/awv184

[97] Zhang H, Wang Y, Liu D, Li J, Feng Y, Lu Y, Yin G, Li Z, Shi T, Wang Z, et al. Carbamate-based N-substituted tryptamine derivatives as novel pleiotropic molecules for Alzheimer’s disease. Bioorg Chem. 2022;125(April):105844. 35594720 10.1016/j.bioorg.2022.105844

[98] Citarella A, Moi D, Pinzi L, Bonanni D, Rastelli G. Hydroxamic acid derivatives: from synthetic strategies to medicinal chemistry applications. ACS Omega. 2021;6(34):21843–21849. 34497879 10.1021/acsomega.1c03628 PMC8412920

[99] Neganova M, Aleksandrova Y, Suslov E, Mozhaitsev E, Munkuev A, Tsypyshev D, Chicheva M, Rogachev A, Sukocheva O, Volcho K, et al. Novel multitarget hydroxamic acids with a natural origin CAP group against Alzheimer’s disease: synthesis, docking and biological evaluation. Pharmaceutics. 2021;13(11):1893. 34834312 10.3390/pharmaceutics13111893 PMC8623418

[100] Kettle AJ, Gedye CA, Winterbourn CC. Mechanism of inactivation of myeloperoxidase by 4-aminobenzoic acid hydrazide. Biochem J. 1997;321(2):503–508. 9020887 10.1042/bj3210503 PMC1218097

[101] Kim H, Wei Y, Lee JY, Wu Y, Zheng Y, Moskowitz MA, Chen JW. Myeloperoxidase inhibition increases neurogenesis after ischemic stroke. J Pharmacol Exp Ther. 2016;359(2):262–272. 27550713 10.1124/jpet.116.235127 PMC5074486

[102] Sodhi RK, Singh N. Defensive effect of lansoprazole in dementia of AD type in mice exposed to streptozotocin and cholesterol enriched diet. PLoS One. 2013;8(7):e70487. 23936214 10.1371/journal.pone.0070487 PMC3729942

[103] Saylam M, Aydın Köse F, Pabuccuoglu A, Barut Celepci D, Aygün M, Pabuccuoglu V. Design, synthesis, and biological activity studies on benzimidazole derivatives targeting myeloperoxidase. Eur J Med Chem. 2023;248(September 2022):115083. 36634456 10.1016/j.ejmech.2022.115083

[104] Regard JB, Harrison TJ, Axford J, Axford L, Lee L, Ren X, Deng L, Reynolds A, Mao J, Liu Q, et al. Discovery of a novel, highly potent and orally bioavailable pyrrolidinone indole series of irreversible Myeloperoxidase (MPO) inhibitors. Biochem Pharmacol. 2023;209(January):115418. 36693437 10.1016/j.bcp.2023.115418

[105] Rodrigues G, De Souza L, Pereira R, Compan R, De Frias B, Amaral M, Freire J, Teles AM, Louback L, Rangel C, et al. Synthesis, in silico, and in vitro evaluation of 7-chloro-quinolines designed as myeloperoxidase inhibitors. J Mol Struct. 2024;1312(P1):138528.

[106] Jantschko W, Furtmüller PG, Zederbauer M, Neugschwandtner K, Lehner I, Jakopitsch C, Arnhold J, Obinger C. Exploitation of the unusual thermodynamic properties of human myeloperoxidase in inhibitor design. Biochem Pharmacol. 2005;69(8):1149–1157. 15794935 10.1016/j.bcp.2005.02.006

[107] Fatima SJ, Prasad DK. Unveiling neuroprotective mechanisms of diosgenin and pterostilbene in diabetes-associated alzheimer’s disease through multi-target molecular docking approach. J Herbmed Pharmacol. 2024;13(4):659–673.

